# Temperature dependence of the dielectric function and critical points of α-SnS from 27 to 350 K

**DOI:** 10.1038/s41598-020-75383-0

**Published:** 2020-10-27

**Authors:** Hoang Tung Nguyen, Van Long Le, Thi Minh Hai Nguyen, Tae Jung Kim, Xuan Au Nguyen, Bogyu Kim, Kyujin Kim, Wonjun Lee, Sunglae Cho, Young Dong Kim

**Affiliations:** 1 Department of Physics, Kyung Hee University, Seoul, 02447 Republic of Korea; 2 Institute of Materials Science, Vietnam Academy of Science and Technology, Hanoi, 100000 Vietnam; 3 Department of Physics and Energy Harvest-Storage Research Center, University of Ulsan, Ulsan, 44610 Republic of Korea; 4 Center for Converging Humanities, Kyung Hee University, Seoul, 02447 Republic of Korea

**Keywords:** Electronic properties and materials, Semiconductors

## Abstract

We report the temperature dependence of the dielectric function *ε* = *ε*_1_ + *iε*_2_ and critical point (CP) energies of biaxial α-SnS in the spectral energy region from 0.74 to 6.42 eV and temperatures from 27 to 350 K using spectroscopic ellipsometry. Bulk SnS was grown by temperature gradient method. Dielectric response functions were obtained using multilayer calculations to remove artifacts due to surface roughness. We observe sharpening and blue-shifting of CPs with decreasing temperature. A strong exciton effect is detected only in the armchair direction at low temperature. New CPs are observed at low temperature that cannot be detected at room temperature. The temperature dependences of the CP energies were determined by fitting the data to the phenomenological expression that contains the Bose–Einstein statistical factor and the temperature coefficient for describing the electron–phonon interaction.

## Introduction

Recently, SnS has been widely investigated due to its p-type semiconductor characteristics and as a promising absorber material for next-generation photovoltaics^1–5^. Its direct bandgap energy is a desirable ~ 1.3 eV with an indirect bandgap near 1.1 eV^6,7^ and a high optical absorption coefficient^8^. Also, SnS-based materials have interesting properties, leading to applications such as high-performance humidity sensors^9^, ion batteries^10^, thermoelectric devices^11^, exciton-driven chemical sensors^12^, and Schottky barrier diodes^13^. They also exhibit valley-selective linear dichroism^14^. To design these devices effectively and enable realistic applications, a knowledge of optical properties of SnS is required.

The complex dielectric function *ε* = *ε*_1_ + *iε*_2_ and refractive index *Ñ* = *n* + *ik* are especially useful for gaining insight into the electronic bandgap structure needed to characterize device performance^15–18^. As a consequence, optical properties have been measured by reflection and absorption^19^, photoreflectance^20^, optical-absorption measurements^21^ on single crystals, UV–Vis-near infrared spectroscopy^22^, photoluminescence^23,24^, and UV–Vis spectrometry^25^ of poly- and nano-crystalline films. Among optical metrology techniques, spectroscopic ellipsometry (SE) is the most powerful for determining dielectric functions and refractive indices of materials^26–30^. It has high accuracy and sensitivity, and does not need to do Kramers–Kronig analysis^31^ to obtain results. Some SE data for single crystal^32^ and thin films^33–35^ have been reported, but only for room temperature.

Single crystal SnS has a highly biaxial anisotropic layered orthorhombic structure (*Pnma* group) with lattice constants of *a* (zigzag) = 4.06 Å, *b* (armchair) = 4.33 Å, and *c* = 11.58 Å, as determined by first principles GW0 Bethe–Salpeter Equation (BSE) theory as reported elsewhere^36^. Lattice constants are close to those previously obtained by electronic band calculations^37–41^. We coordinate *a* and *b* axes to zigzag and armchair, respectively, with the *c*-axis perpendicular to *a*-*b* plane. The biaxial anisotropy is well described in previous literature^32^ but only at room temperature. Room-temperature spectra usually contain enormous contribution of phonon noise, therefore broadening CP structure. Consequently, it is advantageous to obtain data at cryogenic temperatures to reduce thermal noise and to enhance weak features^42–44^. Some optical data are available at low temperature^19^, including temperature dependence^20,21^. However, the spectral energy region is only near-bandgap. There is no systematic analysis of the temperature dependence of *ε* for single-crystal SnS over relatively wide temperature and spectral ranges. Temperature dependences are essential to properly design for extreme environmental applications^45,46^.

Here, we report dielectric-function data for single-crystal biaxial SnS in the spectral range from 0.74 to 6.42 eV from 27 to 350 K. The sample was prepared by the temperature gradient method at 960 °C^36^, as described below. Measurements were done with the sample in ultra-high vacuum conditions. The as-measured pseudodielectric-function data were processed to remove the effects of surface roughness^26,47^ to obtain bulk *ε*_*a*_, *ε*_*b*_, and *ε*_*c*_. At low temperature we detect a strong exciton effect for the armchair direction, which may originate along the Γ-*Y* line in the Brillouin zone. We determined the critical point (CP) energies using 2nd derivative function of *ε* with standard analytic expressions. The results exhibit CPs that cannot be detected at room temperature. The presented information will be useful for engineering devices and understanding the fundamental optical properties of SnS.

## Results and discussion

### SE measurement and analysis at ambient conditions

**Figure 1:**
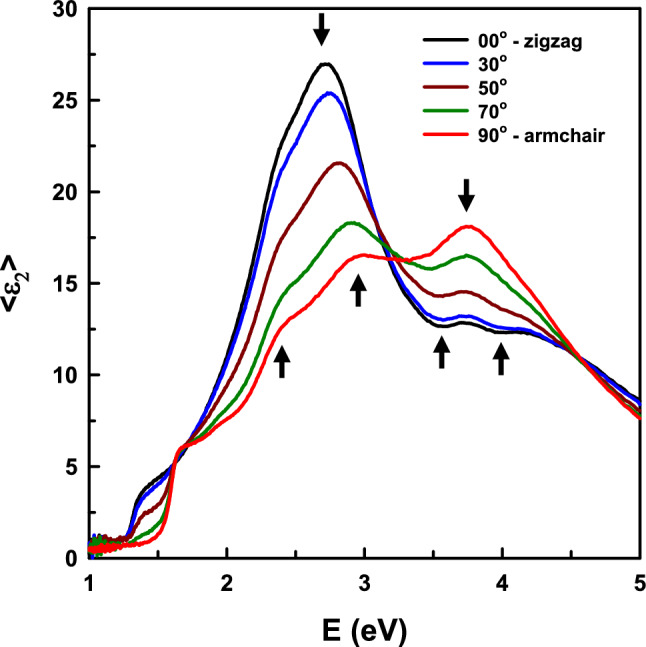
Imaginary parts of dielectric spectra of SnS at room temperature measured by M2000FI at various azimuth angles.

The directions of the crystal axes were determined by measuring the cleavage surface of the sample at 5° azimuth-angle increments using the M2000FI as described in “Methods” section. Results for several angles are shown in Fig. 1. Extreme values of the data at the sharp structures marked by arrows in Fig. 1 are criteria to determine the positions closest to the correct crystal directions. Among the determined positions, the sample is continuously rotated by steps of 1° to accurately identify the directions.

**Figure 2:**
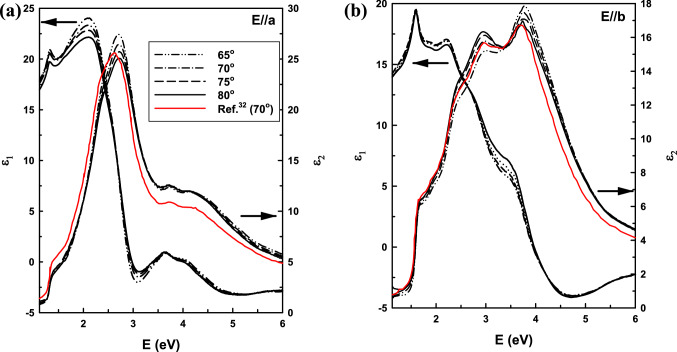
Dielectric spectra of SnS along (**a**) *a* and (**b**) *b* axes at room temperature measured by VASE at various AOI, compared to data previously reported in Ref.^32^.

After the principal directions are determined, the sample was measured at multiple AOIs along the *a* and *b* axes. The results are shown in Fig. 2. Due to anisotropic nature of SnS, the data show strong AOI dependences. The most obvious variations occur near 3 and 4 eV in both directions. While the amplitude of the dielectric function along the *a*-axis at 3 eV decreases by increasing AOI, along the *b*-axis the opposite tendency occurs. Since by increasing the AOI, the contribution of the orthogonal axes to the data is reduced, data obtained at higher AOIs provide a better estimate of the actual dielectric functions of the material along these crystal directions.

Figure 2 also compares our *ε*_2_ room-temperature results to those previously reported by R. E. Banai^32^. Overall, both sets of data are in a good agreement in terms of lineshapes and positions of the main peaks. However, at high eV our data has higher amplitudes, which is one of the criteria to assess data quality. The difference may come from sample preparation, where a better sample with less surface roughness and contamination usually results in below-bandgap values closer to zero and higher values in high eV region (more than 3 eV). Along with measurement at high AOI (80°), our data provide a better estimate of the intrinsic dielectric function of the material.

### SE measurement and analysis at various temperatures

In the first step, the data at 300 K were analyzed using a 3-phase optical model (ambient/a rough surface/SnS). The *ε* of SnS is modeled by the Cauchy model from 0.74 to 1.12 eV. This is well under the direct bandgap of SnS, so it can be considered “transparent” in this range. The dielectric function of the rough surface layer is defined as a mixture of 50% SnS and 50% ambient represented by the Bruggeman EMA model^48^. Data are fitted with *A*, *B*, *C* parameters of the Cauchy model with refractive index *n* = *A*_*n*_ + *B*_*n*_/*λ*^2^ + *C*_*n*_/*λ*^4^ and the thickness of the surface roughness. Our best fits to the SnS data at 300 K yielded the thickness for the rough interface of 3.148 nm, 2.039 nm, and 4.857 nm along the *a*, *b*, and *c* axes, respectively. This is in a good agreement with our AFM results. It is noted that the fitting quality (MSE) is greatly improved by including the thickness of surface roughness as a fitting parameter, from 17.69, 8.89, and 40.86 to 5.13, 2.72, and 1.57 along the *a*, *b*, and *c* axes, respectively. Especially along the *c*-axis, the MSE is improved by a factor of 26, concretely affirming the necessity of involving surface roughness in data processing to obtain the best result.

**Figure 3:**
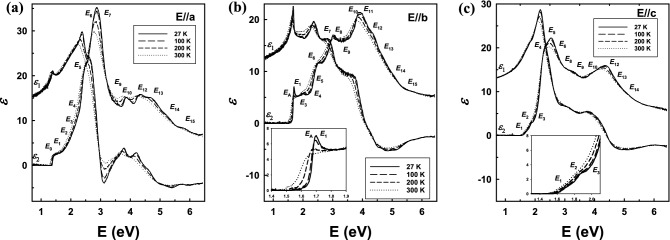
Real and imaginary parts of $\varepsilon$ of SnS at temperatures from 27 to 300 K measured along (**a**) *a*, (**b**) *b*, and (**c**) *c* axes.

In the next step, the point-by-point approach was applied on the proposed three-phase model to extract *ε* of SnS along each axis at various temperatures as shown in Fig. 3. The data shown are at temperatures 27, 100, 200, and 300 K. At low temperature, besides the noticeable blue shift and sharpening of the main CP structures (*E*_7_, *E*_10_, *E*_12_, *E*_13_ along *a*-axis, *E*_1_, *E*_A_, *E*_3_, *E*_6_, *E*_11_, *E*_12_ along *b*-axis, and *E*_5_, *E*_10_ along *c*-axis), various new CPs are distinguished due to reduction of thermal noise in the low temperature data. We note that the change of dielectric function spectrum by temperature results mainly from the CP energy shifts and reduction of broadening of the CP structures, which can be explained by the contributions of thermal expansion and renormalization of band energies due to reduced electron–phonon interactions at low temperature^44,49^. The existence and position of these CPs are carefully examined in next section.

Among the new observations at low temperature, *E*_A_ has not been reported so far in the previous work. The splitting of *E*_1_ and *E*_A_ is clarified in the inset of Fig. 3b. Even though the CP structure of *E*_A_ is very sharp at low temperature, it quickly broadens and disappears with increasing temperature. The *E*_A_ and *E*_1_ CPs are attributed from transitions mainly at a saddle point *M*_1_ along Γ-*Y* direction of the Brillouin zone. This corresponds to the Γ-*X* direction in Ref.^32^.

### CP analysis and determination

The pseudo-dielectric function data < *ε* > were smoothed by the extended Gaussian filtering method^50^ to reduce noise with minimum lineshape distortion. Second order derivatives of *ε* were then fitted to a standard analytic CP expression to obtain CP parameters^51^:

1 $$\frac{{d^{2} \varepsilon }}{{d\omega^{2} }}\begin{array}{*{20}l} { = n\left( {n - 1} \right)A_{{{\text{amp}}}} e^{i\phi } \left( {\hbar \omega - E + i{\Gamma }} \right)^{n - 2} ,} \hfill & { n \ne 0,} \hfill \\ { = A_{{{\text{amp}}}} e^{i\phi } \left( {\hbar \omega - E + i{\Gamma }} \right)^{ - 2} ,} \hfill & {n = 0,} \hfill \\ \end{array}$$

where *A*_amp_, *ϕ*, *E*, Γ, and *n* are the amplitude, phase, threshold energy, broadening, and dimension parameters of a CP, respectively. The exponent *n* =  − 1, − 1/2, 0, and + 1/2 represents excitonic, one-, two-, and three-dimensional CPs, respectively. The CP expression is simultaneously fit to real and imaginary parts of $\frac{{d^{2} \varepsilon }}{{dE^{2} }}$ to obtain the CP parameters. Among the results, we find that the excitonic lineshape (*n* =  − 1) yields the best fit for all CPs.

**Figure 4:**
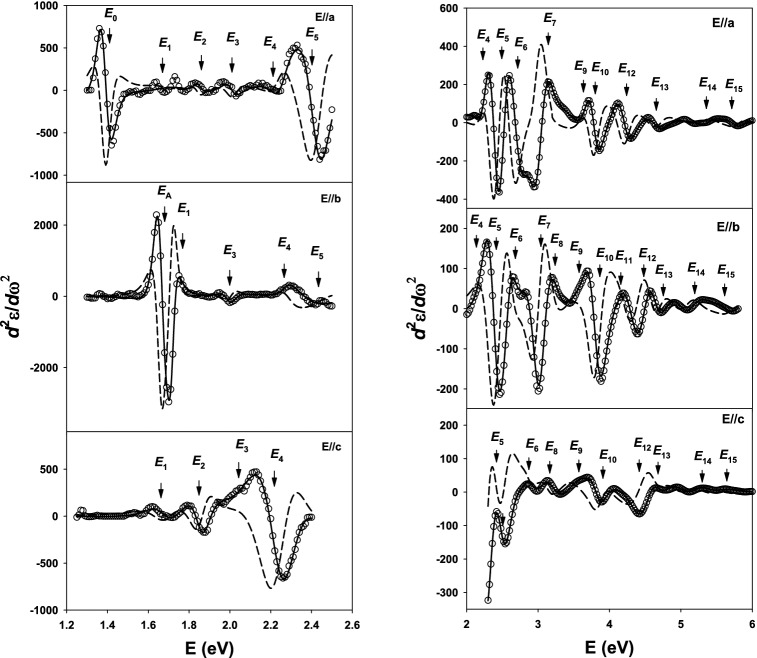
Second energy derivatives of $\varepsilon$ of SnS at 27 K. The best fits are to $\frac{{d^{2} \varepsilon_{2} }}{{dE^{2} }}$ (solid lines) and $\frac{{d^{2} \varepsilon_{1} }}{{dE^{2} }}$ (dashed lines). For clarity, only $\frac{{d^{2} \varepsilon_{2} }}{{dE^{2} }}$ (open circles) is shown, and the number of points is appropriately reduced.

Figure 4 shows the second derivatives of all three axes and their best fits at 27 K. Open circles are calculated $\frac{{d^{2} \varepsilon_{2} }}{{dE^{2} }}$, while the dashed and the solid lines are the best fits of the CP expressions for $\frac{{d^{2} \varepsilon_{1} }}{{dE^{2} }}$ and $\frac{{d^{2} \varepsilon_{2} }}{{dE^{2} }}$, respectively. The calculated lineshapes for $\frac{{d^{2} \varepsilon_{1} }}{{dE^{2} }}$ are not shown. The number of data points for $\frac{{d^{2} \varepsilon_{2} }}{{dE^{2} }}$ are properly reduced for clarity. The figures are divided into two parts, one for low energies and the other for high energies due to relative amplitude of the CPs. In low-CP regions, the CPs are usually sharper, therefore results in higher amplitude. In the derivative spectra, one may identify small structures that are uncertain in the raw data. Along the *a*-axis, *E*_1_, *E*_2_, *E*_3_, and *E*_4_ are minor structures closely spaced from 1.4 to 2.3 eV. These CPs in fact cannot be seen at room temperature. However, at 27 K they become visible in second-derivative data. These CPs might be a “leakage” of other CPs with higher amplitude along other axes like *E*_A_ and *E*_1_ along *b*-axis, *E*_2_ and *E*_4_ along *c*-axis. One realizes that these shared CPs may come from shared transitions in the Brillouin zone or from the low-AOI measurement condition. Realization and explanation of this phenomenon is in preparation for publication at the moment. A closely related picture can be seen in the high energy region where the CPs also share close energy positions along all three axes. Among them, CP structures along the *c*-axis are lower in amplitude and broader in structure in comparison to the CP structures along the other axes. This can be explained by the fact that the atoms are better confined to a more restricted space in the direction perpendicular to the cleavage plane than along *a* and *b* axes.

The CP energies that are the best fits at 27 and 300 K are listed in Table 1 in comparison to Ref.^32^. Our data at 300 K are in a good agreement with data reported at room temperature along all axes. It is noted that the SE experiment in the previous work is only carried out at room temperature. By decreasing the temperature to 27 K, we could observe more CPs, and therefore obtain closer access to intrinsic properties of the material. The identifications of all the CPs are performed by a band structure calculation as presented in the Supplementary Material. We note that even if the dielectric function spectrum has different lineshapes along the different axes, the energies of the same CP structures should have the similar (or same) values since their transitions occur at the same Brillouin zone as shown in the band structure. Therefore, the name of the CP was given by its energy position.

**Table 1:** CP energies at 27 and 300 K compared to data previously reported at room temperature in Ref.^32^ (Errors are smaller than 1% of the fit results).

CP energies (eV)	E//a			E//b			E//c		
	27 K	300 K	Ref.^32^	27 K	300 K	Ref.^32^	27 K	300 K	Ref.^32^
*E*_0_	1.39	1.33	1.31	–	–	1.36	–	–	1.32
*E*_A_	–	–	–	1.66	–	–	–	–	–
*E*_1_	1.67	1.55	1.60	1.71	1.61	1.59	1.67	1.61	1.61
*E*_2_	1.84	–	–	–	–	–	1.86	1.79	1.87
*E*_3_	2.01	–	1.98	2.00	1.90	1.91	2.03	–	–
*E*_4_	2.25	–	–	2.27	–	–	2.24	2.20	2.17
*E*_5_	2.46	2.33	2.34	2.38	2.28	2.28	2.48	2.37	2.43
*E*_6_	2.68	–	2.76	2.60	–	2.64	2.85	–	2.84
*E*_7_	3.02	2.81	3.09	3.02	2.81	2.98	–	–	–
*E*_8_	–	–	–	3.16	–	–	3.15	3.06	–
*E*_9_	3.63	–	3.42	3.56	3.28	3.29	3.45	–	3.29
*E*_10_	3.77	3.65	3.70	3.81	3.71	3.71	3.85	3.74	3.64
*E*_11_	–	–	–	4.20	–	–	–	–	–
*E*_12_	4.21	4.08	4.06	4.46	4.28	4.30	4.53	4.25	4.38
*E*_13_	4.69	4.61	–	4.63	–	–	4.69	–	–
*E*_14_	5.40	–	–	5.11	–	–	5.21	–	–
*E*_15_	5.79	5.36	–	5.71	–	–	5.54	–	–

One of the most noticeable observations is the exciton peak *E*_A_ at 1.66 eV in the spectrum at 27 K along the *b*-axis. As far as we know, this exciton peak has not been reported yet. Therefore, the identification of this CP is ambiguous. We expect that this CP may originate from the same point as *E*_1_ (along Γ-*Y* in the Brillouin zone). In case our assumption is correct, the binding energy of exciton can also be calculated by *E*_1_ − *E*_A_ ~ 50 meV. This value is larger than binding energy of black phosphorus (~ 7.9 meV)^52^ but smaller than that of bulk GeS (~ 300 meV)^53^.

**Figure 5:**
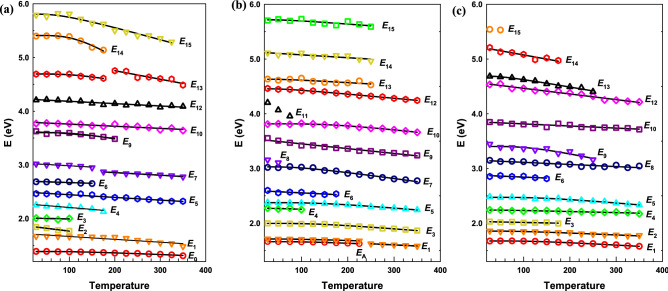
Temperature dependences of the CP energies (open symbols) of SnS and the best fits (solid lines) for CPs of (**a**) *a*, (**b**) *b*, and (**c**) *c* axes.

The temperature dependences of all CPs along all axes are reported in Fig. 5. The open dots are CP energies obtained from the regression analysis and the solid lines are the best fits to either a linear equation or a phenomenological expression that contains the Bose–Einstein statistical factor for phonons^54^. The linear equation is

2 $$E\left( T \right) = E_{{\text{L}}} - \lambda T$$

where *E*_L_ is CP energy at 0 K and − *λ* is the temperature coefficient, *dE*/*dT*. The phenomenological expression is

3 $$E\left( T \right) = E_{{\text{B}}} - a_{{\text{B}}} \left[ {1 + \frac{2}{{e^{{{\Theta }/T}} - 1}}} \right]$$

where *E*_B_, *a*_B_, and Θ, are CP energy at 0 K, strength of electron–phonon interaction, and mean frequency of phonons, respectively. The obtained parameters are listed in Tables 2, 3, and 4 for CPs along the *a*-, *b*-, and *c*-axis, respectively.

**Table 2:** Best-fit parameters for the temperature dependences of CP energies along *a*-axis SnS.

CPs	*E*_B_ (eV)	*a*_B_ (meV)	Θ (K)	*E*_*L*_ (eV)	λ (10^–4^ eVK^−1^)
*E*_0_	1.46 ± 0.02	72 ± 21	385 ± 70	–	–
*E*_1_	–	–	–	1.72 ± 0.02	5.33 ± 0.72
*E*_2_	–	–	–	1.87 ± 0.01	10.57 ± 0.95
*E*_3_	–	–	–	2.01 ± 0.02	1.77 ± 0.30
*E*_4_	–	–	–	2.28 ± 0.02	7.06 ± 0.92
*E*_5_	–	–	–	2.49 ± 0.01	5.02 ± 0.35
*E*_6_	–	–	–	2.70 ± 0.01	2.72 ± 0.93
***E***_**7**_					
27–150 K	–	–	–	3.03 ± 0.01	4.83 ± 0.62
175–350 K	–	–	–	3.03 ± 0.01	4.83 ± 0.62
*E*_9_	4.00 ± 0.02	403 ± 12	413 ± 33	–	–
*E*_10_	–	–	–	3.80 ± 0.01	3.96 ± 0.48
*E*_12_	–	–	–	4.23 ± 0.01	4.34 ± 0.46
***E***_**13**_					
27–175 K	5.05 ± 0.02	354 ± 39	393 ± 55	–	–
200–350 K	–	–	–	5.07 ± 0.02	15.86 ± 0.81
*E*_14_	6.90 ± 0.03	1491 ± 102	425 ± 108	–	–
*E*_15_	6.09 ± 0.03	286 ± 27	236 ± 91	–	–

**Table 3:** Best-fit parameters for the temperature dependences of CP energies along *b*-axis of SnS.

CPs	*E*_B_ (eV)	*a*_B_ (meV)	Θ (K)	*E*_*L*_ (eV)	λ (10^–4^ eVK^−1^)
*E*_A_	1.74 ± 0.02	72 ± 15	357 ± 38	–	–
***E***_**1**_					
27–250 K	1.76 ± 0.02	45 ± 17	287 ± 65	–	–
275–350 K	–	–	–	1.73 ± 0.01	4.05 ± 0.12
*E*_3_	2.16 ± 0.03	162 ± 34	434 ± 51	–	–
*E*_4_	–	–	–	2.28 ± 0.02	2.73 ± 0.20
*E*_5_	2.57 ± 0.01	192 ± 23	475 ± 11	–	–
*E*_6_	–	–	–	2.60 ± 0.01	4.20 ± 0.13
*E*_7_	3.21 ± 0.01	180 ± 45	297 ± 10	–	–
*E*_9_	–	–	–	3.54 ± 0.01	8.80 ± 0.49
*E*_10_	4.13 ± 0.01	313 ± 16	568 ± 65	–	–
*E*_12_	4.53 ± 0.01	70 ± 40	177 ± 12	–	–
*E*_13_	4.68 ± 0.09	48 ± 11	185 ± 32	–	–
*E*_14_	–	–	–	5.13 ± 0.02	5.24 ± 0.30
*E*_15_	5.78 ± 0.13	65 ± 14	196 ± 30	–	–

**Table 4:** Best-fit parameters for the temperature dependences of CP energies along *c*-axis of SnS.

CPs	*E*_B_ (eV)	*a*_B_ (meV)	Θ (K)	*E*_*L*_ (eV)	λ (10^–4^ eVK^−1^)
*E*_1_	1.78 ± 0.02	100 ± 19	388 ± 45	–	–
*E*_2_	1.91 ± 0.02	53 ± 23	276 ± 85	–	–
*E*_3_	–	–	–	2.03 ± 0.01	1.71 ± 0.54
*E*_4_	2.26 ± 0.02	16 ± 21	156 ± 80	–	–
*E*_5_	2.66 ± 0.01	193 ± 56	468 ± 250	–	–
*E*_6_	–	–	–	2.88 ± 0.02	3.58 ± 0.81
*E*_8_	–	–	–	3.15 ± 0.01	3.89 ± 0.47
*E*_9_	3.80 ± 0.03	398 ± 140	383 ± 269	–	–
*E*_10_	–	–	–	3.85 ± 0.01	3.83 ± 0.50
*E*_12_	4.57 ± 0.01	15 ± 18	29 ± 55	–	–
*E*_13_	4.80 ± 0.01	110 ± 25	149 ± 41	–	–
*E*_14_	–	–	–	5.22 ± 0.02	1.52 ± 0.20

In Fig. 5b, the *E*_A_ and *E*_1_ CPs have similar lineshapes and a constant energy gap, which certifies their adjoined transition origins. Even though emergence of excitons in IV–VI materials at low temperatures was also found in GeS^44^, this is the first time an exciton of SnS is realized in a SE measurement as far as we know. We put the energy axis of the CPs in Fig. 5 in a same scale, so that one can recognize the correlation of the CPs along different axes. This correlation relates closely to the origin of the CPs in the Brillouine zone including round shape of *s* orbitals and the anisotropic nature of the *p* orbitals. Detailed explanation of the correlation of the CPs along different axes will be clarified in an upcoming work^55^. By increasing the temperature, some CPs merge with their neighbor CPs to form new CPs and create new trend lines. This behavior can be listed as *E*_6_ and *E*_7_, *E*_13_ and *E*_14_ along *a*-axis, *E*_A_ and *E*_1_ along *b*-axis, *E*_8_ and *E*_9_ along *c*-axis. Besides, there are many CPs can only be observed in very low temperature condition like *E*_2_ and *E*_3_ along *a*-axis, *E*_A_, *E*_4_, *E*_8_ and *E*_11_ along *b*-axis, and *E*_15_ along *c*-axis.

## Conclusions

The anisotropic dielectric responses of SnS are reported and analyzed at different AOI from 65 to 80° in ambient condition and at 68.5° in ultra-high vacuum for various temperatures from 27 to 350 K. The data are obtained by Variable-Angle SE from 1.12 to 6.0 eV at room temperature and by RC2 in the range of 0.74 to 6.42 eV for temperature dependence measurement. By increasing the AOI up to 80°, we approach the intrinsic dielectric functions *ε*_*a*_, *ε*_*b*_, and *ε*_*c*_ along the zigzag, armchair, and c directions of SnS, respectively. By lowering the temperature to 27 K to reduce the electron–phonon interaction, intrinsic properties of the material are revealed including determination of CPs and a separation of overlapping transitions. In this work, many new CPs are discovered as reported above. In particular, the separation of *E*_A_ and *E*_1_ along the *b*-axis has not been observed in SE. All CP energies are extracted precisely by regression analysis of numerically calculated second derivatives of *ε*. Their temperature dependences are determined using a linear equation or a phenomenological expression that contains the Bose–Einstein statistical factor. The results have extended our knowledge of the optical characteristics of SnS, and should be helpful in precise engineering of optoelectronic devices.

## Methods

### Preparation of single crystal α-SnS

Preparation of **s**ingle-crystal α-SnS is described in detail in a previous work^36^. Briefly, single-crystal α-SnS was grown by the temperature-gradient method, where powders of tin (99.8%) and sulfur (99%) were weighted at a molecular ratio of 1:1, respectively. The cleavage surface of the sample contains *a* and *b* axes while *c*-axis is orthogonal to this surface. The sample was carefully cut and polished to prepare a side plane containing *a* and *c* axes.

### AFM

Topographic images of single-crystal SnS were obtained using the contact mode of an AFM (XE-100 Park System). The nominal normal spring constant was 2 N/m and the scan speed was 0.5 Hz.

### SE

Pseudo-dielectric function of SnS were measured by M2000-FI ellipsometer (J. A. Woollam Co., Inc.) on the cleavage plane and the side plane of the sample. The sample was attached on a rotation stage and measured at every 5° rotating angle to determine each crystallographic direction of the sample. The ellipsometer was equipped with a focusing-probe to reduce the size of the beam spot to ~ 100 μm. Data were obtained from 245 to 1000 nm at 1.5 nm intervals and from 1000 to 1664 nm at 3.5 nm intervals. The angle of incidence for the measurement was 70°.

Multiple angle-of-incidence (AOI) measurements were carried out by Variable Angle Spectroscopic Ellipsometry (VASE, J. A. Woollam Co., Inc.). Data were obtained from 1.12 to 6.52 eV at 0.02 eV increments at angles of incidence of 65, 70, 75, and 80°. An attempt to increase the AOI to 85° failed since the sample could not reflect enough light.

For measurements as a function of temperature, a RC2 ellipsometer (J. A. Woollam Co., Inc.) was employed. The SnS sample was isolated in a cryostat system. The sample was mounted on a cold finger and maintained under a base pressure of 10^−8^ Torr to minimize artifacts due to condensation at low temperatures. Temperature was monitored by a silicon-diode thermometer mounted on the same cold finger as the sample. The beam from the light source of the ellipsometer accessed the sample through stress-free fused quartz windows at an AOI of 68.5°. This value is fixed due to design of the cryostat and the ellipsometer.

### Analysis of *ε*

Analysis of the dielectric response of SnS at various temperature was carried out by the WVASE software (version 3.888, J. A. Woollam Co., Inc.). The analysis includes fitting measured data to a three phase model (ambient/surface roughness/SnS) to obtain the thickness of the surface-roughness layer as well as the dielectric function of SnS. The obtained values were used as initial parameters for a point-by-point approach to extract precise values of *ε* of SnS at each temperature.

## Supplementary information

Supplementary Information.
