# Supplementary material for: Temperature dependence of the dielectric function and critical points of α-SnS from 27 to 350 K
Source: Sci Rep. 2020 Oct 27;10:18396. doi: 10.1038/s41598-020-75383-0 (PMC7591561; doi:10.1038/s41598-020-75383-0)
Supplement: Supplementary file 1 — Supplementary Information. [file 41598_2020_75383_MOESM1_ESM.docx]

**Supplementary materials**

**Temperature Dependence of the Dielectric Function and Critical Points of α-SnS from 27 to 350 K**

Hoang Tung Nguyen,^1,2,*^, Van Long Le,^1,2,*^ Nguyen Thi Minh Hai,^3^ Tae Jung Kim^1,4^, Xuan Au Nguyen,^1^ Bogyu Kim,^1^ Kyujin Kim,^1^ Wonjun Lee,^1^ Sunglae Cho^3^ & Young Dong Kim^1^

^1^Department of Physics, Kyung Hee University, Seoul 02447, Republic of Korea

_2_Institute of Materials Science, Vietnam Academy of Science and Technology, Hanoi 100000, Vietnam

^3^Department of Physics and Energy Harvest-Storage Research Center, University of Ulsan, Ulsan 44610, Republic of Korea

^4^Center for Converging Humanities, Kyung Hee University, Seoul 02447, Republic of Korea

Correspondence and requests for materials should be addressed Y.D.K. (ydkim@khu.ac.kr) or T.J.K (tjkim@khu.ac.kr)

^*^These authors contributed equally to this work.

**We assigned the origin of each CP on a band structure calculated by DFT method in ref.^1^. Γ-*X*, Γ-*Y*, and Γ-*Z* directions in the Brillouin zone correspond to *a*, *b*, and *c* crystal axes, respectively.**

**Fig. S1.** Calculated band structure of α-SnS

Reference

1. Le, V. L. Noise Reduction and Investigation of the Anisotropic Dielectric Properties of Single Crystal SnS by Spectroscopic Ellipsometry. (Kyung Hee University, 2020).
